# Editorial Department

**Published:** 1855-03

**Authors:** 


The Twelfth Annual Report of the Managers of the State Lunatic Asylum has been transmitted to the Legislature.

The managers report that

"The Institution committed to our management, has been in successful operation for about twelve years, having been opened for the admission of patients on the 16th of January, 1843. In that period 4,313 patients have been admitted within its walls, and have enjoyed the benefits which it confers. Of this number, 1,789 have been discharged recovered, 55 much improved, 640 improved, 868 unimproved, and 511 have died; leaving 450 patients remaining in the Institution at the close of the year.

"For a full statement of its operations and results during the last year, we refer to the annual report made to us by the superintendent, and which is herewith submitted to the Legislature. From this it will be seen that the whole number of patients under treatment during the year, was 836, of whom 164 were discharged recovered, 42 improved, 115 unimproved, and 65 have died.

"Our former superintendent, Dr. N. D. Benedict, returned about the last of May, from the South, where he had spent the previous winter for the benefit of his impaired health, and on the 10th of June, tendered to the Board of Managers his resignation of the office of superintendent, to take effect on the 30th of that month; and on the 19th day of July, Dr. John P. Gray, who had been the first assistant physician under Dr. Benedict, and had the charge of the Institution during his absence, was unanimously appointed superintendent, in place of Dr. Benedict, resigned. Dr. Edwin H. Van Deusen, second assistant, was appointed first assistant physician.

"On the 19th day of September, Dr. H. S. Swift, acting second assistant, tendered his resignation, to accept the position of resident physician at the New York Hospital; and on the 28th, Dr. John B. Chapin was appointed second assistant physician, to fill this vacancy. Dr. Chapin had previously been connected with the New York Hospital.

"The officers who have had the responsible charge and care of the Asylum, have discharged their arduous duties with great diligence and fidelity, and in a manner which entitles them to the commendation of the Board of Managers."

The success of the treatment seems to have been fully up to the usual average. The daily average number of patients was 440. The whole number treated 836. The number discharged was 386, of whom recovered 164, improved 42, unimproved 115, and died 65. The recoveries were 42.05 per cent. of the admissions. We close our notice of this valuable report with the following extract from the report of the superintendent, Dr. John P. Gray, relative to the treatment of the insane in our county houses:

"With all our facilities, it requires, for the proper care of these persons, an attendant for every six patients. I need not say what treatment they receive in county houses, and yet we transfer annually not less than fifty poor demented patients!

"Many of the superintendents of the poor, especially those who have held the office for several successive years, remove persons belonging to these classes with great reluctance; and during the year several cases of dementia, subject to paroxysms of violence, sent away in former years, have been returned to us from county houses on account of their want of means to take proper care of them. One patient had been for more than a year chained to the floor of his cell, and destitute of clothing and bedding, because *his habits were destructive and filthy.* He had lain upon straw, which was changed two or three times a week, and the person who attended him '*always carried a cowhide, because he always attacked him, and he could only control him by whipping him.*' This man is now quiet and comfortable. Application has recently been made for the return of another, whom the superintendent of the poor states 'has been in chains for months, and is in the most filthy and wretched condition.'

"We have become somewhat habituated to the policy of placing the insane, not likely to recover, in poorhouses among common paupers, and think it no serious wrong; but viewed in a proper light it must be so considered.

"A pauper in health has possession of his intellectual faculties, and is capable of exercising self-control commensurate with his original ability and degree of education, and is held responsible for all his acts. His pecuniary circumstances place him in a poorhouse. An insane person, by reason of disease, is irresponsible and incapable of self government, and requires to be controlled by others. Insanity demands medical advice and special care in all its stages. It afflicts without regard to social position or education, and, generally, cannot be avoided by any ordinary knowledge or forethought; and, in women especially, is often the result of labor in the care of families and in watching over the sick.

"The law provides for the treatment of the insane in an asylum only while their condition is favorable for recovery. Now can it be just that the care of these persons should diminish in the ratio of their increasing helplessness? that the spirit of charity should withdraw further and further as they become more and more dependent on her hand, and when they are utterly helpless leave them to abuse and neglect?" H.

